# Different microRNA profiles reveal the diverse outcomes induced by EV71 and CA16 infection in human umbilical vein endothelial cells using high-throughput sequencing

**DOI:** 10.1371/journal.pone.0177657

**Published:** 2017-05-22

**Authors:** Jie Song, Yajie Hu, Jiaqi Li, Huiwen Zheng, Jingjing Wang, Lei Guo, Ruotong Ning, Hongzhe Li, Zening Yang, Haitao Fan, Longding Liu

**Affiliations:** Yunnan Key Laboratory of Vaccine Research & Development on Severe Infections Disease, Institute of Medical Biology, Chinese Academy of Medical Science and Peking Union Medical College, Kunming, China; Kunming University of Science and Technology, CHINA

## Abstract

Enterovirus 71 (EV71) and Coxsackievirus A16 (CA16) remain the predominant pathogens in hand, foot, and mouth disease (HFMD), but the factors underlying the pathogenesis of EV71 and CA16 infections have not been elucidated. Recently, the functions of microRNAs (miRNAs) in pathogen-host interactions have been highlighted. In the present study, we performed comprehensive miRNA profiling in EV71- and CA16-infected human umbilical vein endothelial cells (HUVECs) at multiple time points using high-throughput sequencing. The results showed that 135 known miRNAs exhibited remarkable differences in expression. Of these, 30 differentially expressed miRNAs presented opposite trends in EV71- and CA16-infected samples. Subsequently, we mainly focused on the 30 key differentially expressed miRNAs through further screening to predict targets. Gene ontology (GO) and pathway analysis of the predicted targets showed the enrichment of 14 biological processes, 9 molecular functions, 8 cellular components, and 85 pathways. The regulatory networks of these miRNAs with predicted targets, GOs, pathways, and co-expression genes were determined, suggesting that miRNAs display intricate regulatory mechanisms during the infection phase. Consequently, we specifically analyzed the hierarchical GO categories of the predicted targets involved in biological adhesion. The results indicated that the distinct changes induced by EV71 and CA16 infection may be partly linked to the function of the blood-brain barrier. Taken together, this is the first report describing miRNA expression profiles in HUVECs with EV71 and CA16 infections using high-throughput sequencing. Our data provide useful insights that may help to elucidate the different host-pathogen interactions following EV71 and CA16 infection and offer novel therapeutic targets for these infections.

## Introduction

Hand, foot, and mouth disease (HFMD) is a widespread viral illness generally occurring in infants and young children, especially those less than 5 years of age[1]. Enterovirus 71 (EV71) and coxsakievirus A16 (CA16), members of the human enterovirus A species within the genus *Enterovirus* of the family Picornaviridae, are the predominant etiological agents of HFMD[2, 3]. Over the past decade, a series of sporadic cases as well as large epidemics associated with HFMD have arisen in the Asia-Pacific region; therefore HFMD has emerged as a serious threat to public health[4]. Although EV71 and CA16 share relatively high nucleotide and amino acid homologies (77% and 89% respectively), the clinical manifestations caused by the two viruses are significantly different[5, 6]. Unlike CA16, which causes milder symptoms that resolve within a few weeks, EV71 infections usually cause severe central nervous system (CNS) complications, such as poliomyelitis-like paralysis, aseptic meningitis, and encephalitis[7, 8]. Thus, prior development of HFMD vaccines has almost exclusively focused on EV71, and 3 successfully developed inactivated EV71 vaccines have been approved by the China Food and Drug Administration (CFDA) since 2015[9, 10]. Although these vaccines are effective in providing protection against EV71 infections, their effectiveness may be limited because they cannot induce highly potent and broad cross-protection against various genotypes of other enteroviruses, including CA16[3, 8]. Furthermore, several severe and fatal cases involving CA16 have recently been reported, and epidemiological surveys indicate that HFMD cases induced by the co-circulation of, co-infection by, or recombination between EV71 and CA16 are increasing[3, 11]. This trend has led to more serious outcomes than would be expected from infection with a single virus. Thus there is an urgent need for a multivalent vaccine against multiple enteroviruses that would help to control enterovirus-related HFMD epidemics. In our previous study, we found that there were significant anti-CA16 neutralizing antibodies in the healthy junior rhesus macaque immunized with the experimentally inactivated CA16 vaccine, but these neutralizing antibodies failed to protect against CA16 infection[12]. Hence, it is our hope that further investigations of the molecular mechanisms of EV71 and CA16 will provide a new strategy for the development of a broad-spectrum HFMD vaccine.

microRNAs (miRNAs) are small, 18- to 25-nucleotide (nt), long single-stranded noncoding regulatory RNA molecules that regulate gene expression at the posttranscriptional level[13]. Through complementary pairing with messenger RNA (mRNA), miRNAs exhibit inhibitory effects by inhibiting translation or stimulating mRNA degradation[14]. A number of recent studies have reported that miRNAs participate in regulating the expression of at least 60% of human genes and play an important role in various physiological processes, including cell proliferation, cell differentiation, hematopoiesis, immune responses, the stress response, and the production of chemokines and cytokines[13]. Therefore, aberrant miRNA expression seems to play a central role in the pathogenesis and progression of several human diseases, especially cancers, neurodegenerative diseases, and cardiovascular disorders[15, 16]. Furthermore, the altered expression of specific miRNAs has been explored in a search for novel biomarkers to aid in the diagnosis of various diseases as well as their progression and the efficacy of their treatment[17]. Additionally, as our knowledge of the role of miRNAs in host-pathogen interactions has advanced, it has been found that viral infections can mediate changes in the expression of cell-specific miRNAs[18]. Such changes represent not only a potential host defense mechanism against infection by efficiently inhibiting viral invasion with minimal clinical consequences but also establish an environment for productive viral infection and/or latency by promoting evasion of the host antiviral immune response or enhancing viral replication and survival in host cells, with the potentially severe outcomes[19]. In other words, these changes in cellular miRNAs may directly contribute to viral pathogenesis, suggesting that in-depth research into cell-encoded miRNA functioninduced by EV71 and CA16 may help to explain why diverse clinical features result from infections caused by these viruses.

Currently, there are several reports indicating that EV71and CA16 can elicit ectopic host miRNA expression profiles in different infected cells, such as human bronchial epithelial (16HBE) cells[20], peripheral blood mononuclear cells (PBMCs) in rhesus monkeys[21], and human rhabdomyosarcoma (RD) cells[22]. However, miRNA biogenesis has unique cell-specific patterns, suggesting that these different miRNA expression profiles would only partially explain the clinical symptoms and might be not involved in the CNS symptoms. EV71 and CA16 are both highly neurotropic viruses that have induced severe CNS complications in patients, and such CNS complications have been recognized as the main causes of fatal HFMD[23]. However, the underlying neuropathogenetic mechanisms of EV71 and CA16 infections have yet to be clearly understood. Two likely routes by which these viruses invade the CNS have been considered: it may be transmitted to the CNS from the blood, across the blood-brain barrier (BBB), or it may enter the CNS through retrograde axonal transport from peripheral nerves[24]. The BBB comprises an interdependent network of brain capillary endothelial cells, endowed with barrier properties, and perivascular cells, containing astrocytes and pericytes that are responsible for maintaining the barrier[25]. Moreover, many neurotropicviruses from the systemic blood circulation, such as Japanese encephalitis virus (JEV)[26], West Nile virus (WNV)[27], can infect the endothelial cells of the BBB, thus disrupting its integrity and eventually entering the CNS. With all this in mind and with the help of high-throughput sequencing technology, we used human umbilical vein endothelial cells (HUVECs), which are often used to build models of the BBB *in vitro*, to investigate alterations in miRNAs due to EV71 and CA16 infection. It is hoped that the results of this study will provide future perspectives regarding the mechanisms underlying EV71 and CA16 neuropathogenesis.

## Materials and methods

### Virus infection and cell line culture

HUVECs purchased from Jennino Biological Technology were cultured in Dulbecco’s modified eagle medium (DMEM) (Gibco, USA) supplemented with 10% fetal bovine serum (FBS) (Gibco, USA), 1% L-glutamine (Gibco, USA), and 1% penicillin/streptomycin (Sigma, USA) in a humidified atmosphere containing 95% air and 5% CO_2_ at 37°C. For *in vitro* virus infection, approximately 1×10^5^ HUVECs per well were plated in 6-well plates and incubated at 37°C under 5% CO2. When HUVECs completely adhered to the 6-well plates, they were infected EV71 (sub-genotype C4, GenBank: EU812515.1) or CA16-G20 strain (sub-genotype B, GenBank: JN590244.1), which were isolated from an epidemic in Fuyang, China in 2008 and from an HFMD patient in Guangxi, China in 2010, respectively. The inoculation quantity of EV71 and CA16 is strictly controlled in lgCCID50 = 5/100μl, that is, the multiplicity of infection (MOI) of EV71 and CA16 is 1. The virus titers of EV71 and CA16 were previously examined in Vero cells and then stored -80℃ in our lab. The cells were collected at 0, 72, and 96 hours post infection (hpi), resuspended in Trizol (TIANGEN, China) and stored at -80°C until used. Cells infected with EV71 and CA16 for 0 hpi were used as controls. Simultaneously, we defined the following experimental groups: EV71-0h, EV71-72h, EV71-96h, CA16-0h, CA16-72h, and CA16-96h. Additionally, the EV71-0h and CA16-0h groups were used for normalization (the normalization value was set to 1), and they were designated as the control. Each group was processed in triplicate and pooled separately for subsequent total RNA extraction.

### RNA extraction and quality control

Samples from the previously mentioned groups were sent to the National Engineering Center for Biochips in Shanghai. miRNA was isolated from each group using a mirVana^TM^ miRNA Isolation Kit (Ambion, USA) in accordance with the manufacturer’s guidelines. RNA purification was carried out after RNA isolation using a miRNeasy Mini kit (Qiagen, Germany) following the manufacturer’s instructions. The quality and integrity of the purified RNA were determined by capillary electrophoresis on an Agilent 2100 Bioanalyzer (Agilent Technologies, USA); only samples with an RNA integrity number (RIN) value greater than 7 were retained for miRNA profile analysis.

### SmallRNA library construction, high-throughput sequencing, and miRNA-seq data analysis

High-throughput sequencing, which possesses lower background noise and higher sensitivity than microarrays, is a powerful tool for transcriptome and noncoding RNA studies, as it can capture almost all expressed known and novel transcripts[28]. Sequencing libraries of the previously mentioned samples were generated using a standard TruSeq Small RNA sample preparation kit (Illumina, USA) according to manufacturer recommendations and then prepared for Illumina sequencing. After Illumina sequencing, the sequence reads went through the data cleaning procedure as described earlier: (1) filtering of low-quality sequences, namely, those with undetermined nucleotides (Ns), a quality score (Q-score) less than 10, or reads shorter than 18 nt using fastx_toolkit-0.0.13.2[29]; (2) removal of the adapter sequences; (3) elimination of noncoding RNAs (e.g., rRNA, tRNA, snoRNA, or snRNA) and repetitive RNAs by mapping clean reads of small RNAs (sRNAs) using Rfam (http://rfam.janelia.org/) and piRNA (http://pirnabank.ibab.ac.in/). The remaining short reads were then mapped to the known miRNA precursors, and the mature miRNAs were deposited in miRBase 19.0 (available online: http://www.mirbase.org/). It is noteworthy that although high-throughput sequencing found novel sequences of miRNAs, we focused mainly on known miRNAs in this study; novel miRNAs have not recently been identified. Finally, 18- to 35-nt lengths of known and novel miRNAs were selected for further analysis. The sequencing data were deposited in the National Center for Biotechnology Information’s Gene Expression Omnibus (GEO) database (www.ncbi.nlm.nih.gov/geo/) and are accessible at series number GSE94551.

### Bioinformatics analysis

**Fig 1:**
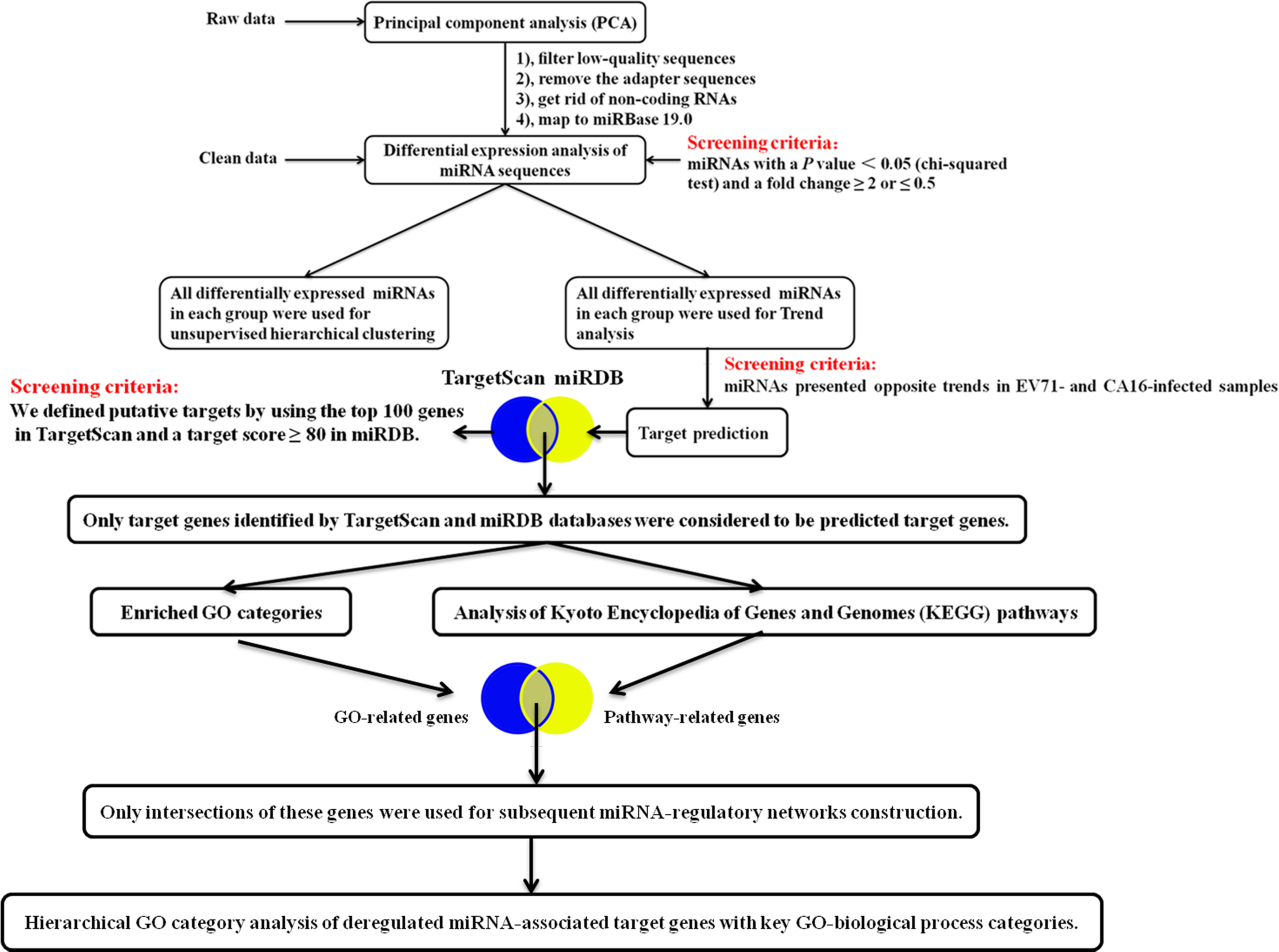
The analysis flow chart of small-RNA sequencing information.

The bioinformatic analysis of the reads aligned to known *Homo sapiens* pre-miRNA in miRBase 19 was performed as previously described[20, 21]. In brief, 7 main aspects, including principal component analysis (PCA), differential expression analysis of miRNA sequences, unsupervised hierarchical clustering, trend analysis, target prediction, and functional enrichment of differentially expressed miRNAs, construction of regulatory networks and hierarchical GO category analysis of deregulated miRNA-associated target genes with key GO-biological process categories were systematically analyzed in this study utilizing the corresponding software. The details of this process are shown in Fig 1.

### Confirmation of differentially expressed miRNAs and miRNA target expression by quantitative RT-PCR (qRT-PCR)

High-throughput sequencing of differentially expressed miRNAs was further verified for 8 randomly selected miRNAs, including 7 known miRNAs, by qRT-PCR using extracted miRNA samples as templates. Additionally, based on an analysis of the predicted targets of the differentially expressed miRNAs, we also randomly chose 20 targets for indirect corroboration of miRNA expression by qRT-PCR. The protocols for qRT-PCR of miRNAs and mRNAs according to the respective manufacturer’s instructions were used as described in our previous study. Relative miRNA and mRNA abundance were determined by normalizing the miRNA of interest to U6 small nuclear RNA (snRNA) and β-actin mRNA, respectively. Then their relative expression was calculated in terms of threshold cycle value (CT) using the 2^-ΔΔCt^ method[30]. Primers were synthesized by Life Technologies (Life Technologies, USA); the names of the genes and their primers are listed in S1 and S2 Tables. The experiments for qRT-PCR detection were carried out at least 3 times.

### Statistical analysis

For the sequencing data, the raw reads obtained from each library were normalized to the number of transcripts per million clean tags (TPMs). For qRT-PCR, the data are presented as the mean ± standard error of the mean (SEM). Statistical analysis was performed using SPSS 18.0 (IBM SPSS, USA). Differences were considered statistically significant for all analyses if *P*<0.05.

## Results

### Comprehensive overview of miRNA sequencing data in HUVECs with infection of EV71 and CA16

**Fig 2:**
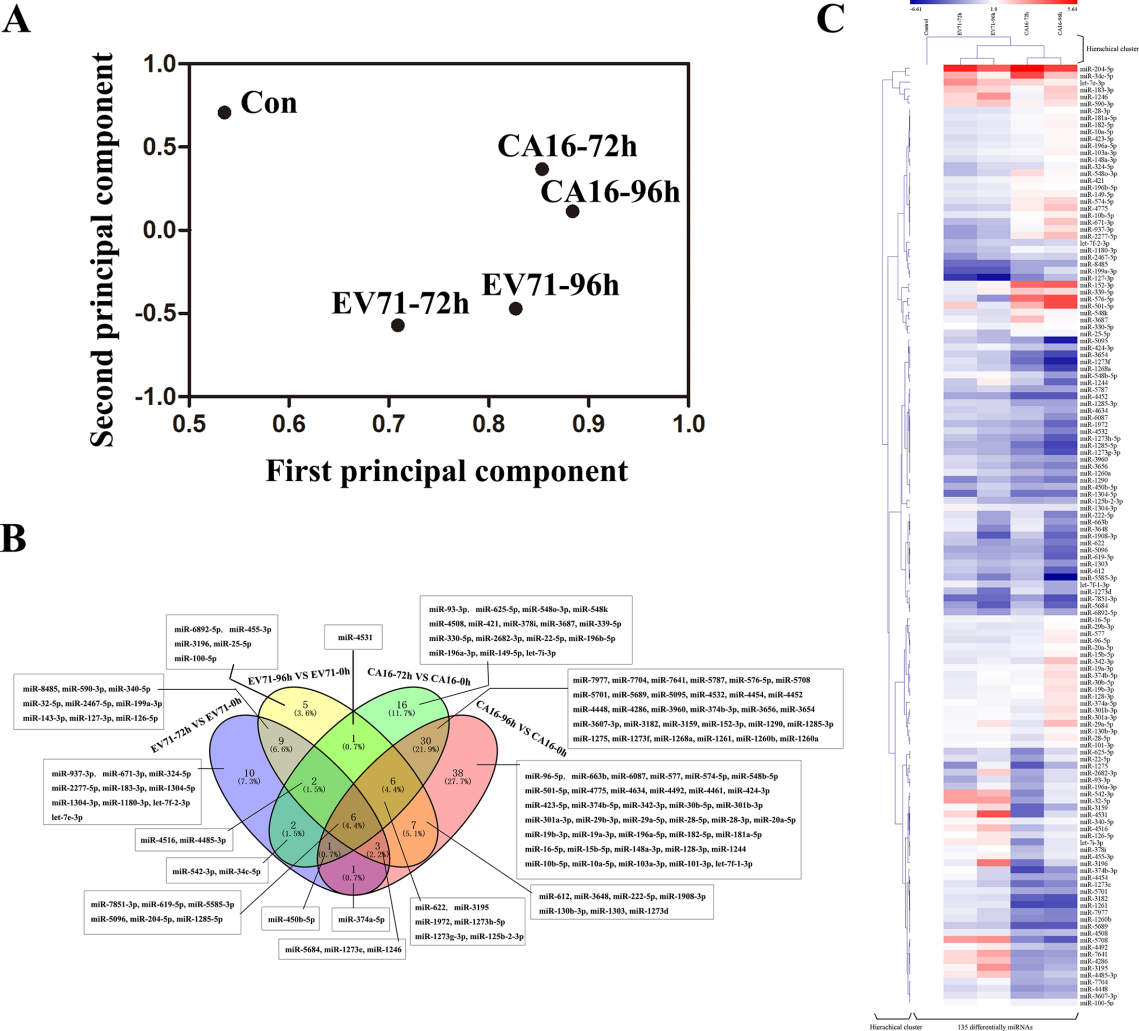
Details of all miRNAs. (A) Principal component analysis (PCA) showing the distribution and clustering of the individual sample groups. Each spot represents a single array. EV71/CA16-infected samples were distinct from the Con sample. (B) Venn diagram representing Known differentially expressed miRNAs.(C) Time- and strain-specific regulation of differential miRNAs during EV71 and CA16 infections. The columns correspond to expression patterns of differentially expressed miRNAs during the EV71 and CA16 infections relative to Con samples at 6 hpi and 12 hpi. Significance was determined using a fold-change threshold of at least 2 and a P value cutoff of 0.05. The intensity of the miRNA expression is indicated in green (lower level of expression) and red (higher level of expression). Dendrograms between samples and between miRNAs are depicted, where the closest branches of the tree represents samples/miRNAs with the most similar expression pattern.

In order to evaluate the effects of EV71 and CA16 infection on miRNA expression in HUVECs, 6 small RNA (sRNA) libraries were constructed and submitted to Illumina sequencing. The details of the resultant sequencing data generation for the subsequent analysis are shown in Table 1. The read classification of different sRNAs types-including miRNA, miscellaneous RNA (misc_RNA), pseudogenes, ribosomal RNA (rRNA), small nucleolar RNA (snoRNA), and snRNA were identified in the samples (S1 Fig), indicating that sRNAs showed dynamic changes in both the EV71- and CA16-infected samples. Additionally, in all libraries, the majority of the sRNAs were 20 to 24nt long, with 22 nt being most abundant, as is typical for Dicer-derived products. Thereafter, the discrete degree of each sample group was examined using PCA (Fig 2A). The results revealed the distribution and clustering of the individual group samples. The Con group and infected groups formed tightly separate clusters, whereas the EV71- and CA16-infected groups were grouped closely together. These results indicate that there was a notable disparity between the EV71-infected and CA16-infected groups.

**Table 1:** Overview of small-RNA sequencing information and subsequent data analysis.

Sample	Raw reads	Clean reads	Adapter-trimmed reads (length > = 18nt)	Reads aligned to known Human pre-miRNA in miRBase 19
EV71-0h	19,633,904	19,399,383	19,013,040	381,861
EV71-72h	19,306,634	19,179,557	18,852,827	575,475
EV71-96h	20,086,058	19,936,799	19,670,311	619,050
CA16-0h	19,076,315	18,857,479	17,538,072	324,901
CA16-72h	20,946,311	20,786,178	20,585,439	380,393
CA16-96h	20,218,869	20,046,884	19,829,142	647,270

Indicated from left to right are the numbers of reads that raw sequencing data, passed quality filtering (clean reads), the numbers of reads that passed both quality filtering, adapter filtering and length filtering (adapter-trimmed reads ≥ 18nt), and the number of reads that could be aligned to known Homo sapiens pre-miRNA in miRBase 19 with perfect matches, respectively.

### Differential miRNA expression profiles associated with EV71 and CA16 infections in HUVECs

To investigate the changes in miRNA expression profiles in HUVECs during EV71 and CA16 infection, all the mappable sRNA sequences were first compared with the currently known *Homo sapiens* miRNAs in the miRBase 19 database; then these miRNA expression patterns following infection were compared with those of the controls. In this study, only those known differentially expressed miRNAs with a threshold *P* value <0.05 and a minimum absolute fold difference of 2.0 between the control and infected data sets are described. As seen in Table 2, after 72 hpi with EV71 treatment, 34 miRNAs were significantly changed, with 14 being upregulated and 20 downregulated. After 96 hpi with EV71 treatment, 39 miRNAs were remarkably changed, with 14 and 25 being up- and downregulated, respectively. After 72 hpi with CA16 challenge, 64 miRNAs were notably altered; 12 miRNAs were upregulated and 52 miRNAs were downregulated. After 96 hpi with CA16 challenge, 92 miRNAs were obviously altered; 36 miRNAs were upregulated and 56 miRNAs were downregulated.

**Table 2:** Significantly differentially expressed miRNAs during the course of EV71 and CA16 infection.

Comparison	Known differentially expressed miRNAs			Rate of differential expression genes (%)
	Total	Up	Down	
EV71-72h vs. EV71-0h	34	14	20	0.0059
EV71-96h vs. EV71-0h	39	14	25	0.0062
CA16-72h vs. CA16-0h	64	12	52	0.0168
CA16-96h vs. CA16-0h	92	36	56	0.0142

Subsequently, the data for all differentially expressed miRNAs were graphed into a Venn diagram (Fig 2B). Common and distinct differentially expressed known miRNAs in response to EV71 and CA16 infection across all time points were displayed. Simultaneously, a total of 135 known differentially expressed miRNAs in all groups were submitted to unsupervised hierarchical clustering to construct a heat map based on the differential expression patterns with log 2 values (infected/control) and fold changes (Fig 2C). A positive log 2 value indicated upregulation and a negative log 2 value indicated downregulation. The heat map exhibited marked discrepancies for the same strain at different times as well as for different strains at the same times following infection. Moreover, the hierarchical clusters of groups were in accordance with the result of PCA. Therefore, these results elucidated that known miRNA expression patterns following infection of EV71 and CA16 are strain- and time-specific.

**Fig 3:**
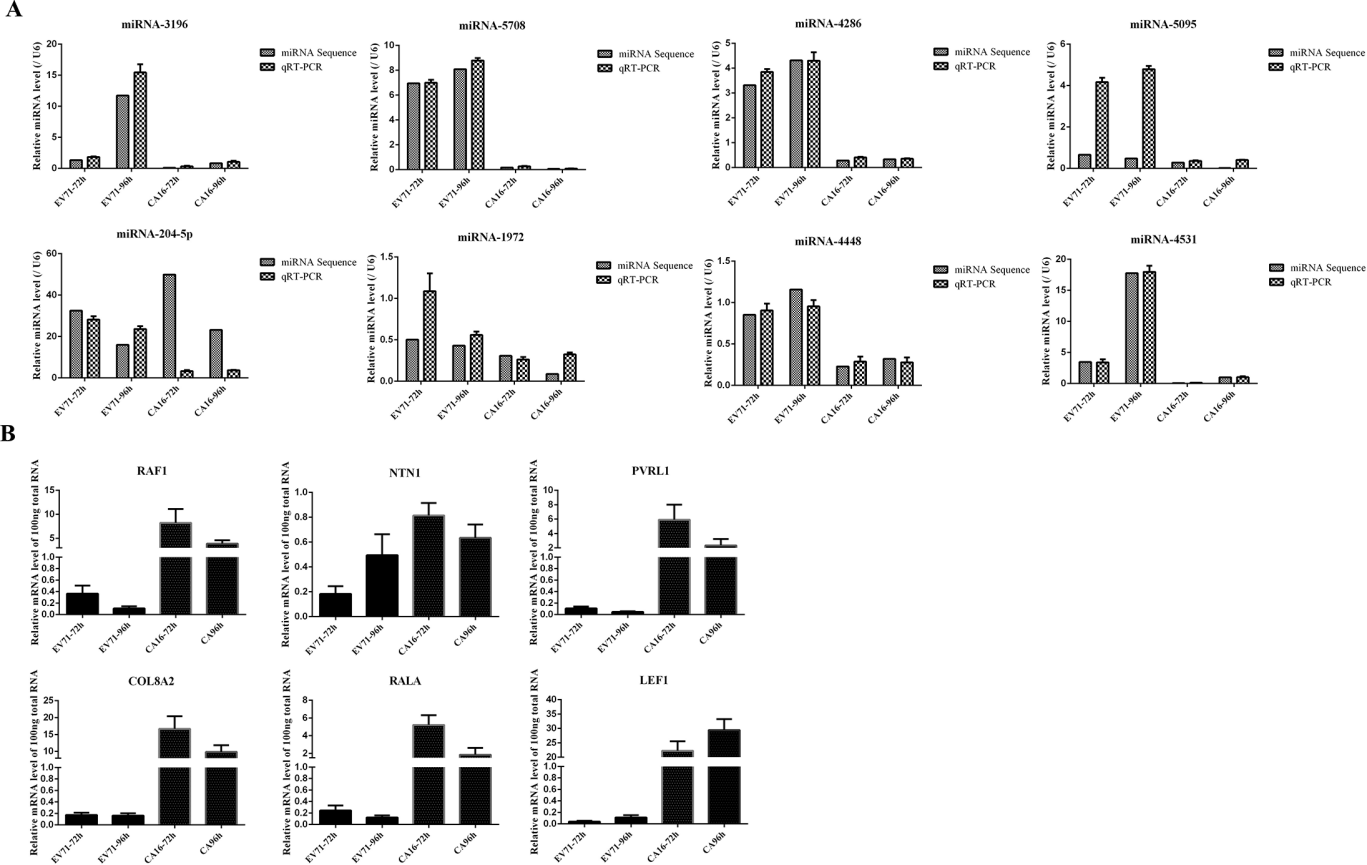
qRT-PCR validation of differentially expressed miRNAs and partial predicted target genes. (A) Eight significantly differentially expressed miRNAs were chosen to perform qRT-PCR validation. Data from qRT-PCR analysis are shown as mean ± SEM (N = 3). N, Times of the experiment was performed. Expression changes are in the same direction as determined by the miRNAs sequencing. (B) Six predicted target genes were detected. The data were calculated as mean ± SEM from triplicates with normalization by β-actin.

Ultimately, to verify the expression patterns of the known miRNAs, 8 differentially expressed miRNAs, namely, miRNA-3196, miRNA-5708, miRNA-4286, miRNA-5095, miRNA-204-5p, miRNA-1972, miRNA-4448 and miRNA-4531 were randomly selected from the high-throughput sequencing for qRT-PCR analysis. Overall, except for miRNA-204-5p, most of the qRT-PCR results of other miRNAs were quite consistent with the results from sequencing data (Fig 3A). Therefore, these results reinforced the reliability of the sequencing data and we concluded that the difference of miRNA-204-5p was primarily due to the limitations associated with the use of qRT-PCR technology.

### Trend analysis of the differentially expressed miRNAs

**Fig 4:**
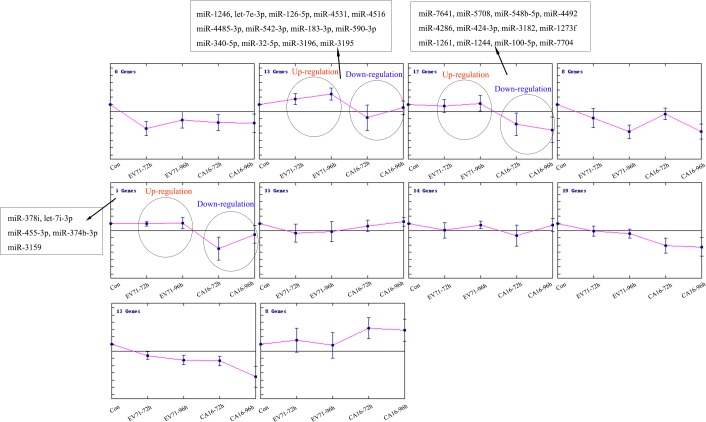
Trend analysis of differentially expressed miRNAs in response to EV71 and CA16 infection over time. The miRNAs that showed opposite expression patterns during the progression of EV71 and CA16 infection are shown in boxes.

Based on previous studies which revealed that there were remarkable differences between EV71 and CA16 infections from clinical observation and experiment data of animal models, we hypothesized that the reasons probably depended on the fact that the host miRNAs induced by CA16 infection might be distinct from those that are induced by EV71 infection. Furthermore, although EV71 and CA16 infections stimulate many common miRNAs, some of the induced miRNAs show opposite trends between the viruses. Trend analysis of miRNA expression dynamics was used to profile the differentially expressed miRNAs and to identify the most probable set of clusters generated in the time series. As illustrated in Fig 4, we observed that 30 known differentially expressed miRNAs were all separately and gradually increased and decreased in EV71 and CA16 infection in the time series, indirectly suggesting that they may greatly contribute to the key distinctions induced by the two viruses. To further narrow down the predominant differentially expressed miRNAs, we defined a screening criterion as follows: if the 2 ratios (EV71-72h/CA16-72h and EV71-96h/CA16-96h) of one of the 30 known differentially expressed miRNAs fell between 0.5 and 2, it was eliminated (Table 3). After applying this criterion, only 27 known differentially expressed miRNAs were regarded as significant.

**Table 3:** Oppositely expressed miRNAs during the course of EV71 and CA16 infection.

miRNAs	EV71-72h (FC)	CA16-96h (FC)	Ratio (EV71-72h/ CA16-72h)	EV71-96h (FC)	CA16-96h (FC)	Ratio (EV71-96h/ CA16-96h)
miR-1246	3.38414545	1.50487685	2.248785638	7.95736516	3.63320466	2.190178065
let-7e-3p	8.6262531	3.41647717	2.52489704	4.31795009	2.50977954	1.720449953
miR-126-5p	2.09121287	0.89907294	2.32596576	2.75713263	1.43982089	1.914913617
miR-4531	3.4753899	0.08122525	42.78706307	17.7691624	1.00391181	17.69992362
miR-4516	2.62407	0.458006	5.729335457	4.54225918	1.08393377	4.190532029
miR-4485-3p	2.70527464	0.3863873	7.0014585	4.22305008	0.68122587	6.199192139
miR-542-3p	6.6355793	0.17082386	38.84456997	4.9348001	1.20469418	4.096309413
miR-183-3p	4.31312655	1.70823859	2.524897041	3.39267507	4.01564726	0.844863816
**miR-590-3p**	**3.31778965**	**2.39153402**	**1.387306066**	**4.16373758**	**2.91134426**	**1.430176993**
**miR-340-5p**	**2.23867019**	**1.29194515**	**1.73279043**	**2.41132278**	**1.68724675**	**1.429146495**
miR-32-5p	6.6355793	0.28470643	23.3067419	6.78535013	1.33854909	5.069182897
miR-3196	1.32711586	0.10830033	12.25403306	11.7201502	0.83659318	14.00937818
miR-3195	2.32245276	0.24403408	9.516919596	8.01905016	0.43024792	18.63820783
miR-7641	3.31778965	0.22476823	14.76093655	6.16850012	0.26418732	23.34896365
miR-5708	6.95077979	0.17082386	40.68974821	8.07689201	0.0649802	124.2977401
miR-548b-5p	1.65889483	0.85411929	1.942228493	2.15897504	0.25097795	8.602249767
miR-4492	1.82478431	0.66431501	2.746866011	2.62161255	0.44618303	5.875643814
miR-4286	3.31778965	0.28470643	11.65337096	4.31795008	0.33463727	12.90337465
miR-424-3p	1.16122638	0.64058947	1.812746593	1.54212503	0.37646693	4.096309413
miR-3182	1.12804848	0.07614242	14.81498108	0.97462302	0.0564214	17.27399554
miR-1273f	1.13752788	0.09855223	11.54238648	0.61685001	0.019306	31.95121341
miR-1261	1.21652287	0.04380099	27.77386766	1.23370002	0.05148266	23.96341006
miR-1244	0.66355793	0.64058947	1.035855196	2.46740005	0.08122525	30.37725397
**miR-100-5p**	**1.76771833**	**1.48347035**	**1.19161015**	**2.01833324**	**1.50058398**	**1.34503185**
miR-7704	1.14131964	0.353229	3.231104057	1.73951703	0.44240182	3.931984385
miR-378i	1.59253903	0.48806817	3.262943869	1.97392004	1.21903577	1.619247016
let-7i-3p	2.65423172	0.17082386	15.53782799	2.77582506	1.20469418	2.304174045
miR-455-3p	1.76948781	0.59131336	2.992470568	2.46740005	1.35141975	1.825783623
miR-374b-3p	1.99067379	0.04641443	42.88911557	0.82246668	0.21512396	3.823222118
miR-3159	1.73769495	0.08122525	21.39353154	3.23075681	0.37646693	8.581781146

Three miRNAs eliminated are highlighted in bold. FC, fold change.

### Gene ontology (GO) and pathway analysis of predicted targets of differentially expressed miRNAs

**Fig 5:**
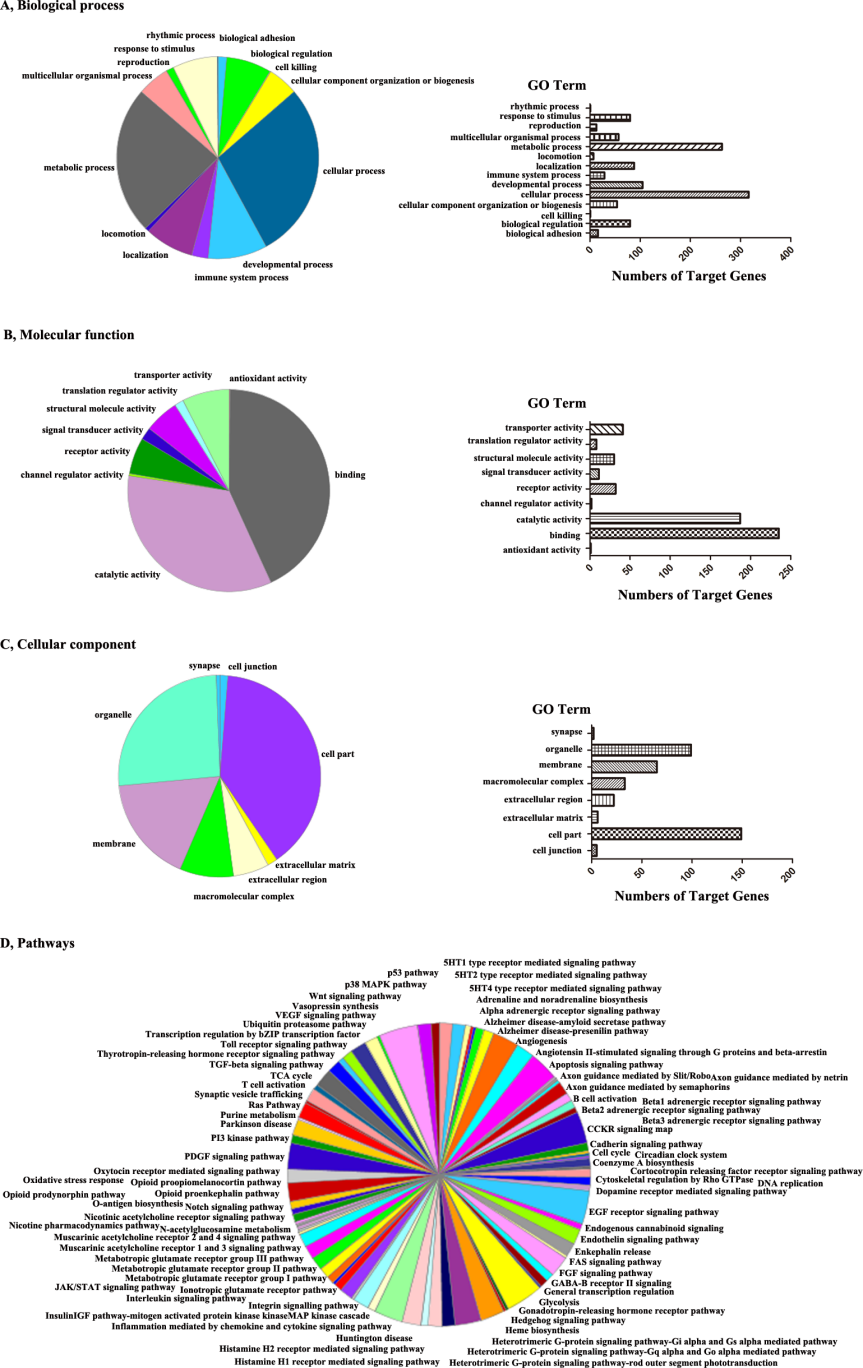
Gene ontology enrichment terms and KEGG pathway analysis of the predicted targets of differentially expressed miRNAs. The DAVID web-based tool was used to analyze (A) biological process, (B) molecular function and (C) cellular component. GO functional enrichment annotations exhibit in pie charts of the left panel. Bars indicate the number of GOs annotated as unique GO terms and are presented in the right panel. (D) There are 85 pathways in total, as depicted in the pie chart.

To gain insight into the possible regulatory roles of the 27 known differentially expressed miRNAs in EV71 and CA16 infections, we used the TargetScan and miRDB programs to predict the potential targets of these miRNAs[31, 32]. We found that 2,305 genes were predicted to be potential targets with TargetScan, whereas 3,449 genes were predicted to be potential targets with miRDB (S2 Fig). Then GO was introduced into the analysis to reveal functions significantly associated with the predicted target gene candidates of the altered miRNAs. Predicted target functions were classified into 3 core categories: biological process (Fig 5A), molecular function (Fig 5B), and cellular component (Fig 5C). Biological process included ontology rhythmic process, response to stimulus, reproduction, multicellular organismal process, metabolic process, locomotion, localization, immune system process, developmental process, cellular process, cellular component organization or biogenesis, cell killing, biological regulation, and biological adhesion. The molecular function category included transporter activity, translation regulator activity, structural molecule activity, signal transducer activity, receptor activity, channel regulator activity, catalytic activity, binding, and antioxidant activity. The cellular component category included synapse, organelle, membrane, macromolecular complex, extracellular region, extracellular matrix, cell part, and cell junction. Afterward, the predicted targets genes were subjected to the KEGG pathway enrichment analysis using the PANTHER Classification System. As shown in Fig 5D, 85 pathways were identified, suggesting that these signaling pathways are regulated by the differentially expressed miRNAs during viral infection. Among these pathways, many were intimately correlated with immune regulation (e.g., the apoptosis signaling pathway, B-cell activation, interferon-gamma signaling pathway, interleukin signaling pathway, Toll receptor signaling, T-cell activation, etc.), adhesion signaling (e.g., cadherin signaling pathway, cytoskeletal regulation by Rho GTPase, Wnt signaling pathway, etc.) and nervous system function (e.g., axon guidance mediated by netrin, synaptic vesicle trafficking, etc.), which suggested that these alterations involved different immune responses, cell adhesion, and neurological symptoms induced by EV71 or CA16 infection.

### Complex regulation network construction of miRNA-targets and the miRNA-GO and miRNA-pathways

**Fig 6:**
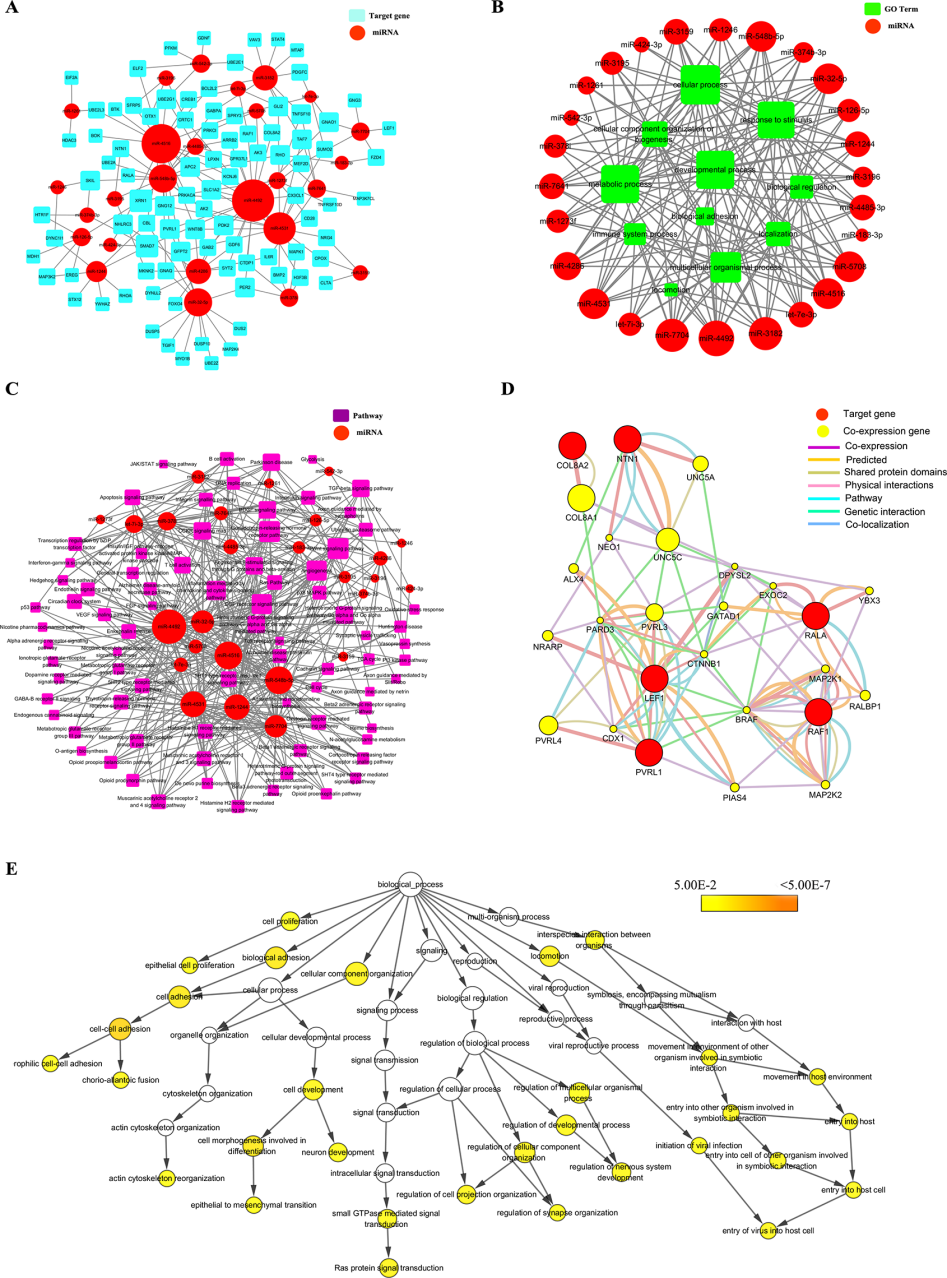
Complexity of the miRNA-regulation networks after infection with EV71 and CA16. miRNAs are depicted as a red colored node at (A), (B) and (C). (A) miRNA-targets network. The targets are displayed by light blue color rounded rectangles. (B) miRNA-GOs network. GO term nodes are depicted as green color rounded rectangles. Edges in the network represent inhibitory effect of miRNAs on GOs. (C) miRNA-pathways network. Purple rectangle-shaped nodes denote pathways. Edges indicate a negative correlation between miRNA and pathways. (D) Co-expression network were constructed by adhesion-related targets. Red nodes are target genes and yellow nodes are co-expression genes. Genes with bigger size are more centralized in the network and have a stronger capacity of modulating adjacent genes. Different color lines mean the different interactions between these genes. (E) Hierarchical GO categories of genes involved in adhesions. A *P* value cutoff of 0.05 was used to determine significantly enriched GOs. The color gradient of the yellow nodes, from light to dark, displays the P value, from high to low (the lower the P value, the greater the GO significance level). White nodes are no significant.

To gain a deeper understanding of the regulatory functions of miRNAs, we built miRNA regulatory networks to elaborate the interactions among miRNAs and genes, GOs, and pathways. First, to confirm the key target genes, we looked for intersections of related genes in the results of our GO and pathway analyses. A total of 91 common target genes were determined by Venn diagram; these are listed in S3 Fig. Next, 26 known differentially expressed miRNAs, 11 GOs involved in biological processes, and 81 pathways were identified by refining the correlations among the 91 target genes and the miRNAs, GOs, and pathways. Consequently, by integrating these correlations, interaction networks between miRNAs and targets (Fig 6A), miRNAs and GOs (Fig 6B), and miRNAs and pathways (Fig 6C) were established. Among the 3 networks, 1 miRNA often regulates more than 1 of the target genes, GOs, and pathways, whereas a target gene, GO, or pathway is also often regulated by more than 1 miRNA.

### Co-expression network analysis, hierarchical GO category analysis, and detection of target genes involved in key biological processes

In this part, we specifically looked into the hierarchical GO categories of the deregulated miRNA-associated targets with the biological adhesion derived from the previously described biological process analysis. In this biological process category produced by the GO analysis, 8 known differentially expressed miRNAs and 6 targets were found to participate in biological adhesion (Table 4). Moreover, these targets were used to build a co-expression network (Fig 6D), which contained adhesion molecules (e.g., PVRL3, PVRL4, and CTNNB1, etc.), nervous regulation molecules that participated in axon extension and cell migration during neural development (e.g., UNC5C and UNC5A), and cellular structure formation–related molecules involved in the establishment of cell polarity (e.g., PARD3), among others. These targets and co-expression genes may provide new information regarding the underlying distinct mechanisms caused by EV71 and CA16 infection. In addition, in order to investigate the inner functions of the genes involved in our gene network on the ontology level, these targets were also submitted to a hierarchical GO categories analysis. The results of this pointed to statistically significantly enriched GO terms among the given gene list based on their hypergeometric probability. In Fig 6E, the 6 targets are seen to be significantly enriched in 5 key biological processes, including cell proliferation (e.g., epithelial cell proliferation), cell adhesion (e.g., cell-cell adhesion, actin cytoskeleton reorganization, etc.), signal transduction (e.g., small GTPase–mediated signal transduction and Ras protein signal transduction), nervous regulation (e.g., regulation of synapse organization, regulation of nervous system development, etc.), and interplay between host and virus (e.g., initiation of viral infection, entry into host cell, etc.). Hence, according to these findings, we proposed that 8 known differentially expressed miRNAs may indirectly mediate these GOs, which might harbor significance and/or contribute to the pathogenesis and biochemical characteristics of HFMD patients afflicted with EV71 or CA16 infection.

**Table 4:** Biological adhesion process related to miRNAs and their target genes.

miRNAs	Target genes
let-7i-3p, miR-3195, miR-32-5p, miR-4492 miR-4516, miR-548b-5p, miR-5708, miR-7704	RAF1, NTN1, PVRL1 COL8A2, RALA, LEF1

Subsequently, we chose the 6 targets to further measure and confirm the miRNA sequencing data. In general, the expression levels of these miRNAs and their targets presented an inverse relationship based on the regulatory mechanisms of the miRNAs. Except for NTN1, the expression levels of target genes were inversely correlated with the expression patterns of the miRNAs regulating these target genes (Fig 3B).

## Discussion

EV71 and CA16, as the main pathogens responsible for worldwide HFMD outbreaks, can cause severe neurological complications and death[1]. Although 3 inactivated EV71 vaccines have already shown substantial protective effect against EV71-HFMD and EV71-related diseases in clinical trials, the current lack of an effective vaccine to treat HFMD induced by other enteroviruses, including CA16, highlights the importance of developing vaccines to prevent the transmission of the other causative agents[9]. Thus, further investigations identifying the factors that cause the different manifestations of EV71 and CA16 infection are still urgently needed and may provide new strategies for the development of more efficacious vaccines to prevent HFMD outbreaks. In the current study, we identified a total of 135 differentially expressed miRNAs across different time points following EV71 and CA16 infection; of these, both common and unique miRNAs were identified, indicating that miRNA expression in the context of these viruses has strain- and time-specific tendencies. Moreover, since the 6 common miRNA, namely, miR-7851-3p, miR-619-5p, miR-5585-3p, miR-5096, miR-204-5p, and miR-1285-5p simultaneously participated in both EV71 and CA16 interactions with host cells. It is possible that 1 or 2 of them could serve as potential candidates for the antiviral therapy of HFMD. It has recently been estimated that about 60% of human genes may be subjected to miRNA regulation through the degradation of target mRNAs or the suppression of mRNA translation following specific binding to target mRNA, and the roles of miRNAs in pathogen-host interactions have received increasing attention[14, 17]. A growing body of reports indicates that viral infections can remodel cellular miRNA expression, which in turn influences cellular microenvironment and metabolism and eventually leads to viral pathogenesis[33]. For example, the influenza virus notably changes cellular miR-106b, miR-124, and miR-1254 expression in favor of influenza replication by regulating human protease genes (such as ADAMTS7, CPE, DPP3, MST1, and PRSS12), which can cause clinical outcomes ranging from mild upper respiratory infections to severe pneumonia[34]; respiratory syncytial virus (RSV) obviously enhances let-7f expression in the host, which likely contributes to delayed viral clearance and inhibits let-7i and miR-30b expression, thus promoting viral replication[35]. Moreover, these altered miRNAs appear to exacerbate the severity of disease. Hence it is no surprise that abnormal miRNA expression is intimately associated with the development and progression of various infectious diseases[19]. Additionally, evidence from different researchers is accumulating that some miRNAs are involved in the infectious cycle of CA16 or EV71; for instance, 3 key miRNAs (miR-545, miR-324-3p, and miR-143) can be used to distinguish EV71 infection from CA16 infection in patients with HFMD[36]. Furthermore, elevated expression of the circulating miR-876-5p is a specific response to severe EV71 infections[37], and miR-432* regulates the replication of CA16 in RD cells[38]. Another study has reported a dramatic reduction in EV71 replication when DGCR8 (an essential cofactor for miRNA biogenesis) was knocked down prior to EV71 infection, suggesting that EV71 might utilize the host’s miRNAs to enhance viral replication[39]. Collectively, our discovery of the specific miRNA expression patterns that are produced during EV71 and CA16 infection in HUVECs provides powerful insights into the different mechanisms of viral pathogenesis regulated by miRNAs. Following, these findings further elucidate the key disparities between EV71 and CA16 infection.We further isolated 30 differentially expressed miRNAs that displayed opposite expression trends. The functions of some miRNAs among these 30 have been studied by other researchers. For example, miR-183-3p was validated as a potent prognostic marker for lung adenocarcinoma in female nonsmokers[40], and declining expression of let-7e-3p was associated with cell cycle arrest and the inhibition of proliferation of cervical cancer cells[41]. High endogenous and circulating miR-1246 modulated by the direct upstream regulator octamer 4 (Oct4) was shown to be a novel diagnostic marker as well as a tool for therapeutic intervention in hepatocellular carcinoma (HCC) by cooperatively driving Wnt/β-catenin signaling activation[42]. In addition, miRNA-1246 was found to be a target gene of p53 in the progression of various cancers, including cervical, colorectal, esophageal, hepatic, and pancreatic cancers[43–45]. It was also found that the expression of miR-1246, which was increased in EV71-infected cells (in agreement with our findings), could suppress the expression of the disc-large homolog 3 (DLG3), known to contribute to neurological disorders[46]. In addition, the miR-4492 was significantly upregulated in EV71-infected RD cells, consistent with our results[47]. However, the specific roles of these differentially expressed miRNAs in our study of EV71 and CA16 infection remain largely unknown and need further exploration.

In order to better understand the potential molecular mechanisms of these differentially expressed miRNAs that occur during EV71 and CA16 infection, we carried out GO and pathway analysis of the predicted target genes of the 30 differentially expressed miRNAs identified in trend analysis. Using KEGG pathway analysis and GO annotation for target genes can offer a comprehensive understanding of the target genes at th**e** pathway, molecular, cellular, and biological levels. The observed changes in biological processes (especially related to immune system processes and biological adhesion), molecular function (especially with reference to binding), cellular components (especially linked to cell junctions) and pathways (especially associated with immune pathways, such as apoptosis signaling pathways, the Toll-receptor signaling pathway, T-cell activation, interleukin signaling, inflammation mediated by chemokine and cytokine signaling, B cell activation, the JAK/STAT signaling pathway, etc., and adhesion pathways, such as the cadherin signaling pathway, cytoskeletal regulation by Rho GTPase, the Wnt signaling pathway, etc.) suggested that these differentially expressed miRNAs might play important roles in immune and adhesion responses in HUVECs infected with EV71 and CA16. Nevertheless, the success of pathogens in entering host cells largely depends on their invasion strategies, including impeding the activation of the host immune system, destroying intercellular junctions, triggering excess apoptosis, and so on[48]. Furthermore, biochemical and genetic studies have clarified that miRNAs, as key regulators, participate in the regulation of immune function, such as antigen presentation (e.g., miR-155), T-cell receptor signalling (e.g., miR-181a), Toll-like receptor signalling and the ensuing cytokine responses (e.g., miR-146), etc.[49], and cell-cell contact, such as urothelial permeability (e.g., miR-199a-5p), etc[50]. Additionally, it has been demonstrated that EV71 infection can affect the p38 MAPK signaling pathway, which may be associated with the secretion of inflammatory cytokines and host-cell apoptosis; these mechanisms may be implicated in inflammation of the CNS and disorders such as encephalitis or meningitis[51]. Similarly, in our study, the p38 MAPK signaling pathway was also changed by pathway enrichment analysis. Moreover, the predicted target genes were also enriched into specific neuronal cellular components, such as synapses, and neuronal function-related pathways, such as axon guidance mediated by Slit/Robo, netrin, or semaphorins, etc. Thus we hypothesized that the differing pathogeneses of EV71 and CA16 infections are partly attributable to the regulatory roles of the oppositely expressed miRNAs involved in the immune response, intercellular junctions, and neurological symptoms that might lead to the malfunction of host cells or favor the viral life cycle. Next, the associations of the identified miRNAs with target genes, GOs, pathways and co-expression genes were further visualized by creating regulatory networks.

It is known that the BBB is composed of microvascular endothelial cells, pericytes, and astrocytes, comprisinga complex physical barrier through tight cell-cell interactions and having a critical role in protecting against invading pathogens and establishing mechanical strength[25]. Once the integrity of the BBB is disrupted, CNS pathogenesis is facilitated through increasing viral invasion and spread. For example, West Nile virus (WNV) infection of the brain’s microvascular endothelial cells (HBMVEs) significantly increases leukocyte adhesion to the HBMVE monolayer and transmigration across the HBMVE, suggesting that WNV causes destruction of the BBB. This improves leukocyte infiltration into the brain and fosters neuroinflammation[27]. Dengue virus (DENV) infection, for example, changes the localization of the tight junction proteins Zonula occludens (ZO-1) and Claudin-1 in mouse brain endothelial cells (MBECs), and this process is closely associated with a decrease in transendothelial resistance, an increase in macromolecule permeability, and an increase in the paracellular passing of free virus particles in MBECs, indicating that the elevated permeability of the BBB allowed the entry of virus into the nervous system and caused the neurological manifestations[52]. EV71 and CA16 are also highly neurotropic viruses, potentially causing varying degrees of neurological complications[24]. In the present study, therefore, we focused mainly on the biological adhesion results derived from the GO analysis to perform a more in-depth hierarchical GO category analysis. Our results demonstrate that the 8 differentially expressed miRNAs implicated in biological adhesion were consistently upregulated following EV71 infection and downregulated following CA16 infection. Furthermore, owing to the negative regulation that miRNAs exert on their target genes, it was proposed that an inverse correlation exists between the miRNAs and the corresponding GOs of their target genes. Notably, the functions of biological adhesion induced by CA16 infection were significantly activated, whereas they were inhibited by EV71 infection. These results of hierarchical GO category analysis showed that there are 5 significant differences, as follows: cell proliferation (e.g., epithelial cell proliferation), cell adhesion (e.g., cell-cell adhesion, actin cytoskeleton reorganization, etc.), signal transduction (e.g., small GTPase-mediated signal transduction and Ras protein signal transduction), nervous regulation (e.g., regulation of synaptic organization, regulation of nervous system development, etc.) and interplay between host and virus (e.g., initiation of viral infection, entry into the host cell, etc.).These effects as induced by EV71 infection are more susceptible to dysregulation than they are in the context of CA16 infection. Moreover, most of the target genes linked to biological adhesion for the 8 differentially expressed miRNAs described previously were declined during EV71 infection but overexpressed during CA16 infection, as validated by qRT-PCR. These results further demonstrate that the cell-cell contacts of HUVECs may be disturbed during EV71 infection, indicating impairment of the BBB. Taken together, we hypothesize that the neuropathological manifestations of EV17 and CA16 infection may be mediated by the 8 differentially expressed miRNAs. Additionally, in our previous study, miR-4516, involved in biological adhesion, was reduced and elevated in the EV71 and CA16 infection of human bronchial epithelial (16HBE) cells[20]. According to the previous speculation, miR-4516 may be the key factor causing the destruction of 16HBE cells in CA16 infection and ultimately leading to the repeatedly occurring phenomenon of CA16 infection and its clinical features, which rarely happens following EV71 infection (unpublished data). Nevertheless, in this study, miR-4516, also discovered in biological adhesion, was increased and decreased in the EV71 and CA16 infection of HUVECs, respectively. Therefore it was postulated that the altered miR-4516 generated in the HUVECs subjected to EV71 and CA16 infections may initiate different degree impairment of HUVECs, thus creating a relatively permissive environment for viral invasion into the brain and contributing to the different outcomes of EV71 and CA16 infections in the CNS.

In conclusion, for the first time, this study has demonstrated, by small RNA sequencing technology and bioinformatics approaches, that EV71 and CA16 infections result in specific miRNA expression patterns in HUVECs. More studies are needed to dissect the relevant roles of miRNAs in viral pathogenesis. GO and pathway analysis of the predicted targets of the differentially expressed miRNAs were screened from trend analysis and provided comprehensive information about the discrepant biological functions and pathways involved EV71 and CA16 infection. Furthermore, the construction of detailed regulatory networks alsorevealed a global perspective for exploring miRNA-mediated mechanisms. Consequently, it is worth noting that the 8 known differentially expressed miRNAs related to biological adhesion identified in this study are the key miRNAs that modulate cellular junctions in HUVECs and compromise the permeability barrier of HUVECs; these activities may play a role in the dissemination of virus in the CNS.

## Supporting information

S1 Table Primers used for qRT-PCR confirmation of miRNA sequencing. (DOCX)

S2 Table Sequences of target primers used for qRT-PCR assays. (DOCX)

S1 Fig Bar chart summarized the different classes of sRNAs in the samples. Different types of sRNAs, including miRNA, miscellaneous RNA (misc_RNA), pesudogene, ribosomal RNA (rRNA), small nucleolar RNA (snoRNA) and small nuclear RNA (snRNA), were identified in the samples.
(TIF)

S2 Fig Venn diagram of the key target genes number in TargetScan analysis and miRDB analysis. The common putative targets between TargetScan and miRDB analysis are 731.
(TIF)

S3 Fig Map of overlapped target genes between GO and pathway analysis. 91 target genes were observed and listed on a box.
(TIF)
