# Supplementary material for: Different microRNA profiles reveal the diverse outcomes induced by EV71 and CA16 infection in human umbilical vein endothelial cells using high-throughput sequencing
Source: PLoS One. 2017 May 22;12(5):e0177657. doi: 10.1371/journal.pone.0177657 (PMC5439704; doi:10.1371/journal.pone.0177657)
Supplement: S1 Table — (DOCX) [file pone.0177657.s001.docx]

| **miRNAs** | **Primer sequences** |
| --- | --- |
| miR-3196 | 5’-CGGGGCGGCAGG-3’ (sense) |
| miR-5708 | 5’-TGAGCGACTGTGCCT-3’ (sense) |
| miR-4286 | 5’-AGACCCCACTCCTGGT-3’ (sense) |
| miR-5095 | 5’-AGTTACAGGCGTGAACCA-3’ (sense) |
| miR-204-5p | 5’-CGCAGTTCCCTTTGTCATC-3’ (sense) |
| miR-1972 | 5’-CAGGCCAGGCACAGT-3’ (sense) |
| miR-4448 | 5’-CAGGGCTCCTTGGTCT-3’ (sense) |
| miR-4531 | 5’-CGCAGATGGAGAAGGCT-3’ (sense) |
