# Supplementary material for: Different microRNA profiles reveal the diverse outcomes induced by EV71 and CA16 infection in human umbilical vein endothelial cells using high-throughput sequencing
Source: PLoS One. 2017 May 22;12(5):e0177657. doi: 10.1371/journal.pone.0177657 (PMC5439704; doi:10.1371/journal.pone.0177657)
Supplement: S2 Table — (DOCX) [file pone.0177657.s002.docx]

| **Genes** | **Primer sequences** |
| --- | --- |
| COL8A2 | 5’- CCACGGAACTAAGTAGGGTCCCAA -3’ (sense)  5’- GACACCTGCAACTCAAATCCCAT -3’(anti-sense) |
| RAF1 | 5’- AACTTTGCTCGGAAGACGTT -3’ (sense)  5’- AGCCACAAGTCTGACATCGAA-3’(anti-sense) |
| NTN1 | 5’- AGGCACTTTACCCACATGCTC -3’ (sense)  5’- CCACCAATTTGCAGCATGACC-3’(anti-sense) |
| PVRL1 | 5’-CTTGGCCTGCATCGTCAACTACCACA-3’ (sense)  5’-GCAGGTACCAGTTGCCATCAAACCC-3’(anti-sense) |
| RALA | 5’- TTGAGAAGTAACTGTCCGCTA -3’ (sense)  5’- TCATTCAAACATAACTGGTGCT-3’(anti-sense) |
| LEF1 | 5’- CCCTCATCCAGCTATTGTAACACC-3’ (sense)  5’- TCAGCAACGACATTCGCTCT-3’(anti-sense) |
