# Supplementary figures and images for: Different microRNA profiles reveal the diverse outcomes induced by EV71 and CA16 infection in human umbilical vein endothelial cells using high-throughput sequencing
Source: PLoS One. 2017 May 22;12(5):e0177657. doi: 10.1371/journal.pone.0177657 (PMC5439704; doi:10.1371/journal.pone.0177657)

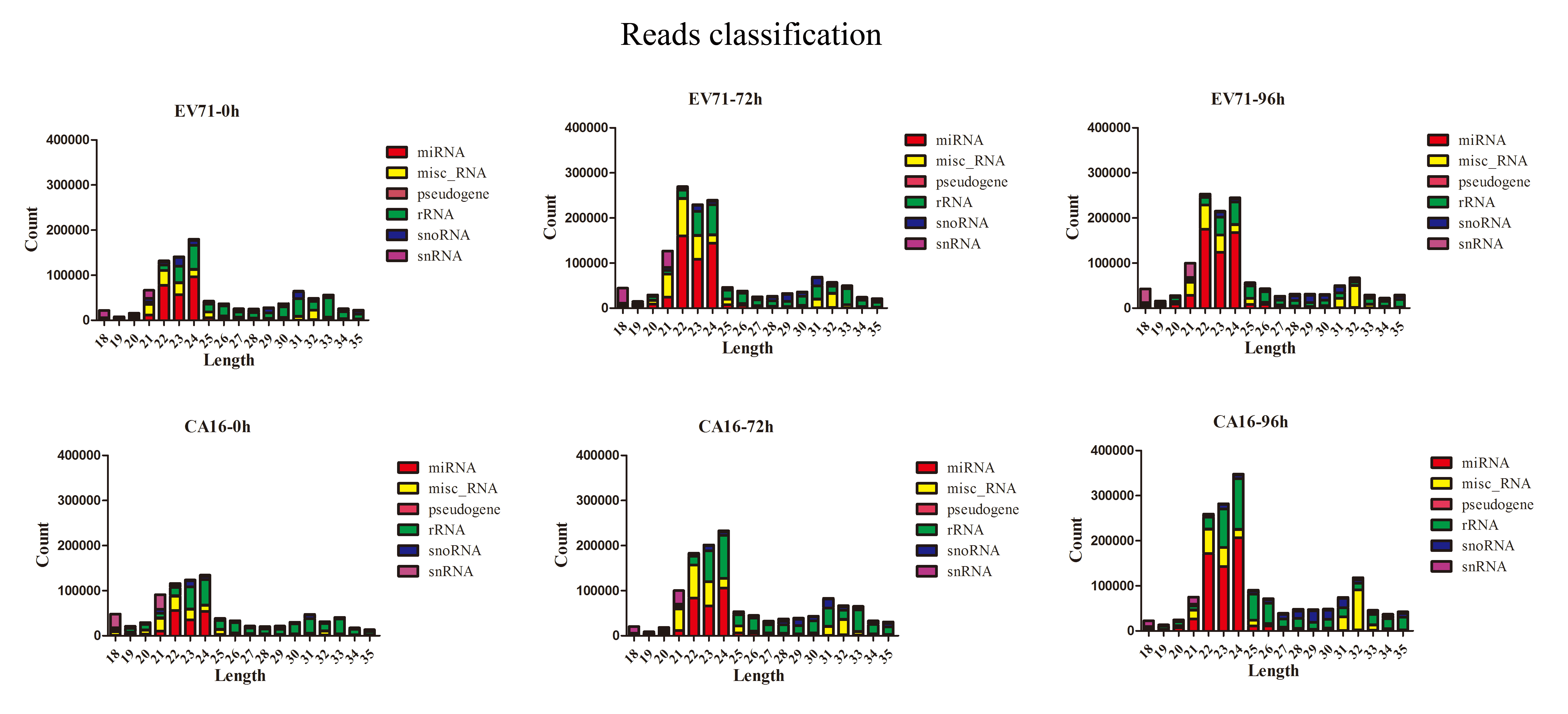

Supplement: S1 Fig — Different types of sRNAs, including miRNA, miscellaneous RNA (misc_RNA), pesudogene, ribosomal RNA (rRNA), small nucleolar RNA (snoRNA) and small nuclear RNA (snRNA), were identified in the samples. (TIF) [file pone.0177657.s003.tif]

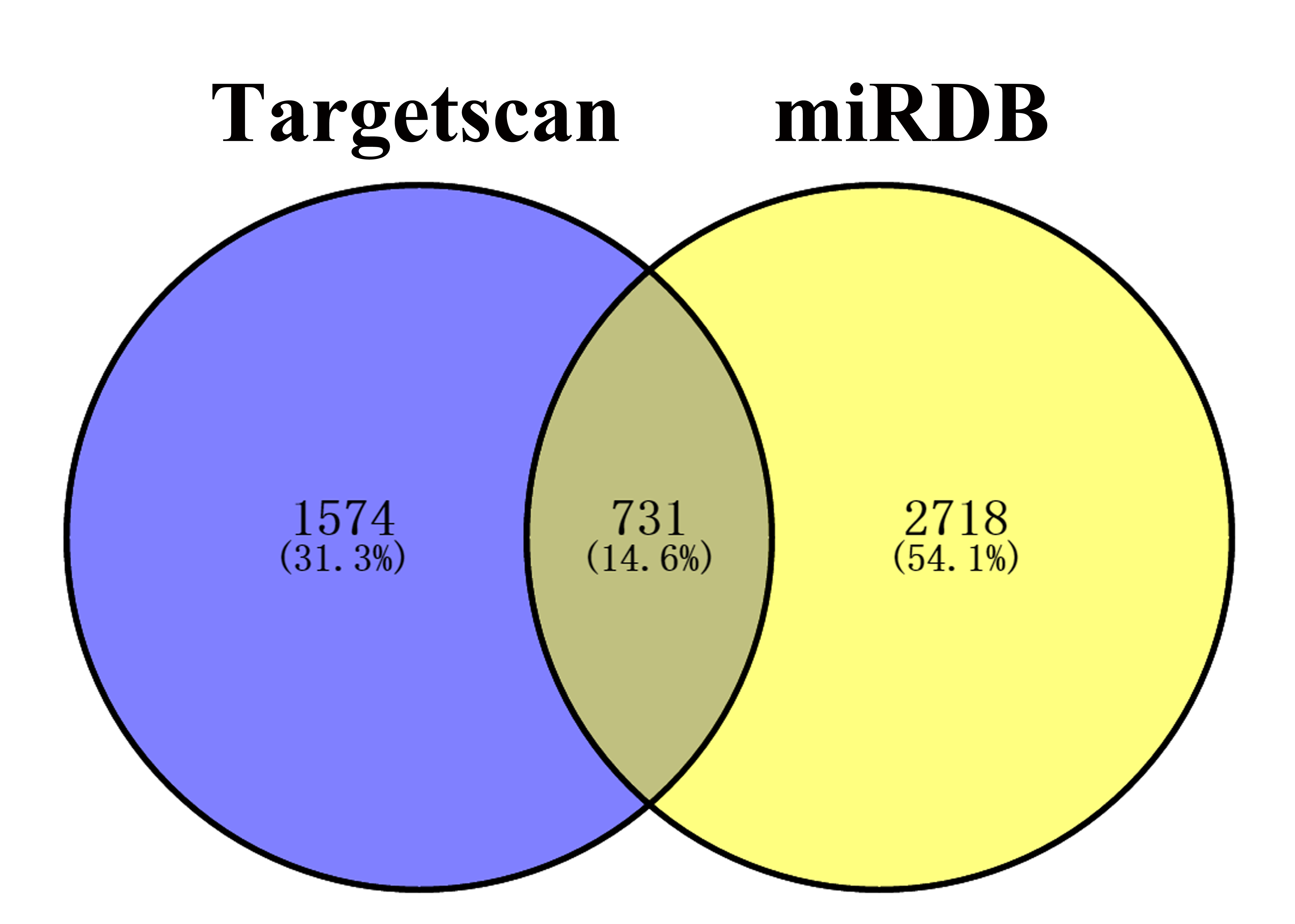

Supplement: S2 Fig — The common putative targets between TargetScan and miRDB analysis are 731. (TIF) [file pone.0177657.s004.tif]

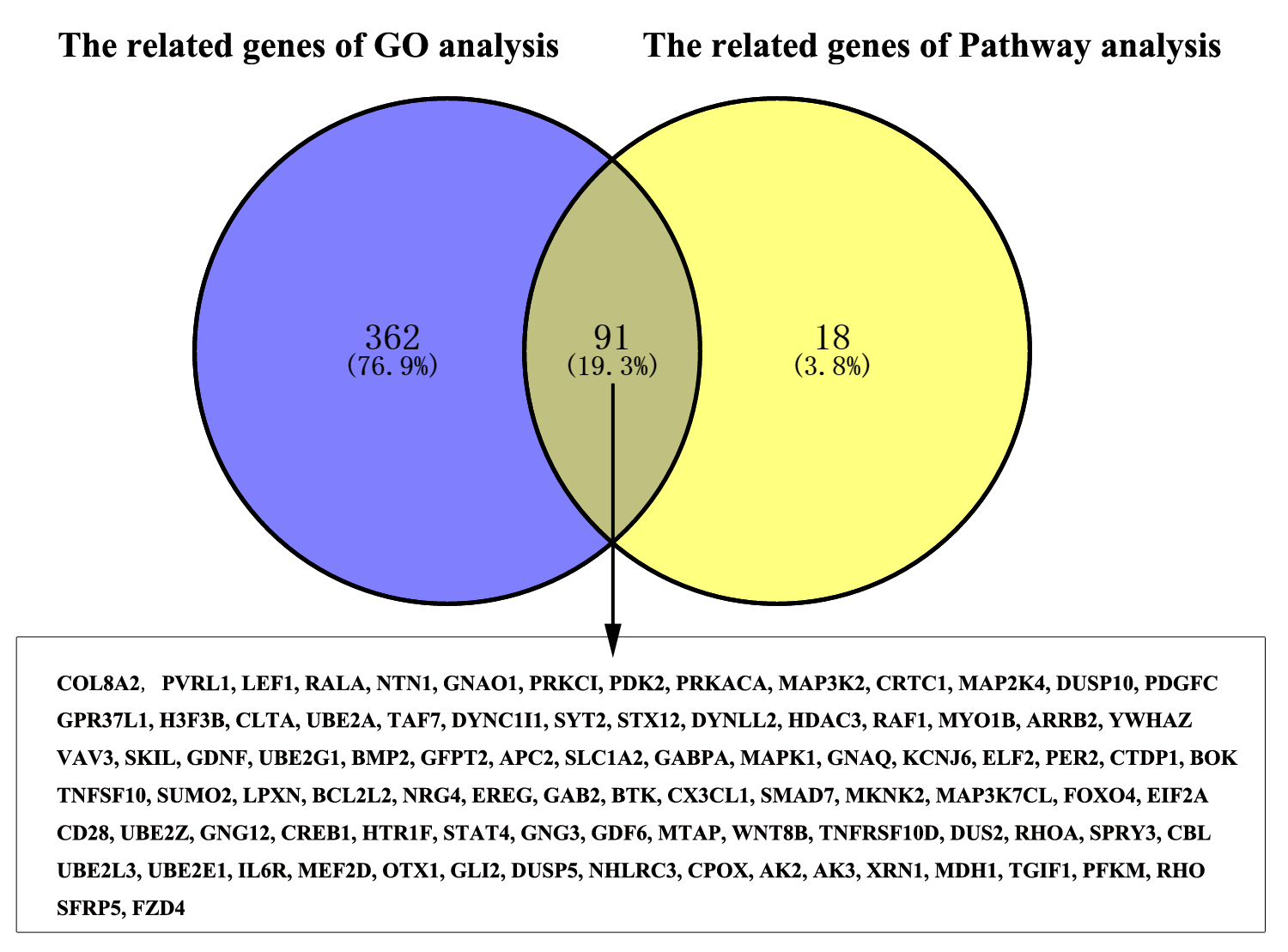

Supplement: S3 Fig — 91 target genes were observed and listed on a box. (TIF) [file pone.0177657.s005.tif]
